# Optimizing Vital Signs in Patients With Traumatic Brain Injury: Reinforcement Learning Algorithm Development and Validation

**DOI:** 10.2196/63847

**Published:** 2025-07-03

**Authors:** Hongwei Zhang, Mengyuan Diao, Sheng Zhang, Peifeng Ni, Weidong Zhang, Chenxi Wu, Ying Zhu, Wei Hu

**Affiliations:** 1 Department of Critical Care Medicine, Affiliated Hangzhou First People's Hospital, School of Medicine, Westlake University, 261 Huansha Road, Hangzhou, 310006, China, 86 13634164536; 2 Department of Critical Care Medicine, Ruijin Hospital, Shanghai Jiao Tong University, School of Medicine, Shanghai, China; 3 Department of Critical Care Medicine, The Fourth College of Clinical Medicine, Zhejiang Chinese Medicial University, Hangzhou, China

**Keywords:** traumatic brain injury, reinforcement learning, temperature, mean arterial pressure

## Abstract

**Background:**

Traumatic brain injury (TBI) is a critically ill disease with a high mortality rate, and clinical treatment is committed to continuously optimizing treatment strategies to improve survival rates.

**Objective:**

This study aims to establish a reinforcement learning algorithm (RL) to optimize the survival prognosis decision-making scheme for patients with TBI in the intensive care unit

**Methods:**

We included a total of 2745 patients from the Medical Information Mart for Intensive Care (MIMIC)–IV database and randomly divided them into a training set and an internal validation set at 8:2. We extracted 34 features for analysis and modeling using a 2-hour time compensation, 2 action features (mean arterial pressure and temperature), and 1 outcome feature (survival status at 28 d). We used an RL algorithm called weighted dueling double deep Q-network with embedded human expertise to maximize cumulative returns and evaluated the model using a doubly robust off-policy evaluation method. Finally, we collected 2463 patients with TBI from MIMIC III as an external validation set to test the model.

**Results:**

The action features are divided into 6 intervals, and the expected benefits are estimated using a doubly robust off-policy evaluation method. The results indicate that the survival rate of artificial intelligence (AI) strategies is higher than that of clinical doctors (88.016%, 95% CI 85.191%‐90.840% vs 81.094%, 95% CI 80.422%‐81.765%), with an expected return of 28.978 (95% CI 28.797‐29.160) versus 27.092 (95% CI 24.584‐29.600). Compared with clinical doctors, AI algorithms select normal temperatures more frequently (36.56 °C to 36.83 ℃) and recommend mean arterial pressure levels of 87.5‐95.0 mm Hg. In external validation, the AI strategy still has a high survival rate of 87.565%, with an expected return of 27.517.

**Conclusions:**

This RL algorithm for patients with TBI indicates that a more personalized and targeted optimization of the vital signs is possible. This algorithm will assist clinicians in making decisions on an individualized patient-by-patient basis.

## Introduction

Traumatic brain injury (TBI) constitutes a major cause of mortality and morbidity among patients with trauma, with an estimated 69 million new cases globally each year. In the United States, data from the Centers for Disease Control and Prevention reported 288,000 TBI-related hospitalizations in 2014, resulting in 56,800 fatalities [1]. In China, the mortality rate attributable to TBI stands at approximately 13 per 100,000 individuals [2], while in France and South Africa, the rates are 5.2 and 80.73 per 100,000, respectively [3]. TBI encompasses a spectrum of clinical presentations, from transient concussions to profound comas. Management of TBI is multifaceted, encompassing prehospital care, emergency department interventions, surgical procedures, and intensive care unit (ICU) management. ICU treatment specifically involves comprehensive strategies including hemodynamic monitoring, intracranial pressure (ICP) control, temperature regulation, thromboprophylaxis, gastrointestinal ulcer prevention, seizure prophylaxis, and nutritional support [4]. Ongoing research is essential to refine and optimize ICU management protocols for patients with TBI.

Machine learning (ML) and artificial intelligence (AI) hold significant potential for advancing clinical decision-making processes. Prior research has demonstrated the utility of ML in various domains, including the management of sepsis [5,6], weaning from acute respiratory distress syndrome [7], and optimizing mechanical ventilation settings [8,9]. The aim of reinforcement learning (RL) is to use interactions with an environment to find actions that maximize reward. Guided trial-and-error search and delayed feedback are key aspects that distinguish RL from other ML methods [10]. Compared with other ML methods, advantages of RL include strong self-learning, high adaptability, long-term reward optimization, and sequential decision-making. RL addresses the balance between exploration and exploitation through its reward mechanism. RL adjusts its policy to learn the optimal action in the context of sparse feedback, avoiding heavy reliance on labeled data. This advantage is particularly evident in task-oriented dialogue systems or complex task management, where RL can learn to respond to indirect or delayed feedback. RL holds immense potential in the ICU, particularly in personalized treatment, real-time decision-making, and complex monitoring tasks. It can assist physicians in formulating and optimizing treatment plans, improving the quality and safety of critical care for patients [11]. Patients in the ICU often present with complex and rapidly changing conditions, and standardized treatment protocols may not meet individualized needs. RL models can learn from historical data about how different patients respond to treatments, allowing for dynamic adjustments to the treatment plan and the creation of personalized strategies for each patient.

Despite its promise, there is a paucity of AI research specifically targeting the management of TBI in the ICU. To address this gap, we used a novel algorithm, the weighted dueling double deep Q-network with embedded human expertise (WD3QNE), to optimize treatment strategies for TBI. The WD3QNE algorithm advances traditional double deep Q-networks with dueling networks and dueling deep Q-network methods by incorporating a target Q-value function with adaptive dynamic weights, thereby enhancing estimation accuracy [12]. Furthermore, it integrates clinical expertise to improve the performance of RL in clinical decision-making.

## Methods

### Data Sources and Data Processing

The study cohort comprised patients with TBI aged 18 years and older, as identified in the Medical Information Mart for Intensive Care (MIMIC)–IV database using *International Classification of Diseases‌*, *Ninth Revision*, codes 800, 801, 803, 804, 850‐854, and *International Classification of Diseases‌*, *Tenth Revision*, code S06. Only the first ICU admission for each patient was included, with the dataset divided into a training set (80%) and an internal validation set (20%). External validation was performed using data from the MIMIC-III database. Collected patient variables included age, Glasgow Coma Scale, Systemic Inflammatory Response Syndrome, Sequential Organ Failure Assessment (SOFA) score, heart rate, respiratory rate, FiO2, pCO2, SpO2, pO2, pO2/FiO2 ratio, temperature, mean arterial pressure (MAP), white blood cell count, hemoglobin, platelet count, activated partial thromboplastin time, prothrombin time, international normalized ratio, glucose, total bilirubin, lactate, creatinine, aspartate aminotransferase, alanine aminotransferase, blood urea nitrogen, pH, base excess, bands, potassium, sodium, calcium, magnesium, chloride, bicarbonate, and urine output. Data were collected for the first 72 hours post-ICU admission, with a time step of 2 hours. For multiple measurements within a 2-hour interval, the mean value was used, except for the Glasgow Coma Scale (minimum value) and urine output (summed). Missing data were initially forward-filled, and remaining gaps were imputed using the k-nearest neighbors algorithm. Continuous variables were normalized to a range of −1 to 1. The experiments were conducted in a Python 3.9 environment using the PyTorch framework. All computations were performed on a personal computer equipped with a 2.60 GHz Intel Core i5-11400F CPU and 16GB of RAM.

### Ethical Considerations

MIMIC data has undergone strict deidentification processing by the Massachusetts Institute of Technology, and all patient information cannot be traced back to personal identity. Moreover, MIMIC data collection has obtained exemption from the Institutional Review Board of the Massachusetts Institute of Technology for informed consent from patients. Researchers MD and HZ have completed the CITI Program ethical examination certification on the PhysioNet platform, submitted an application through PhysioNet, and signed a data usage agreement (certification numbers 1630201 and 13402134).

### RL Algorithm Overview

#### Markov Decision Process

We simulated the health trajectories and clinical decisions of patients in the ICU using a Markov decision process (MDP), which is a continuous interaction process between the agent and the environment. By defining 5 elements—state space (*S*), action space (*A*), state transition probability (*P*), reward function (*R*), and discount factor (*γ*)—the interaction process between the agent and the environment is transformed into a computable model [13]. In 2010, Alagoz et al [14] proposed using MDP to solve the problem of sequential clinical treatment under uncertainty, and subsequently more researchers have focused on clinical decision analysis in RL [15].

The specific analysis process is as follows: the intelligent agent selects action *A_t_* based on the current state *S_t_*; For state *S_t_* and action *A_t_*, MDP obtains *R_t_* and *S_t_* based on the reward function and state transition function, and feeds them back to the agent. The goal of an intelligent agent is to maximize the accumulated reward obtained. The function by which an intelligent agent selects an action from the set of actions *A* based on its current state is called strategy *π*. The strategy *π*(*a|s*)*=P*(*A_t_=a|S_t_=s*) is a function that represents the probability of taking action “*a”* after the input state “*s”*. When a strategy is a stochastic policy, it outputs a probability distribution of actions in each state, and then samples based on this distribution to obtain an action. In MDP, the state value function *V^π^*(*s*) based on policy *π* is the expected return that can be obtained by following policy *π* starting from state “*s”*; In addition, due to the existence of actions, an additional action value function *Q^π^* (*s,a*) based on policy *π* is defined to represent the expected return obtained by performing action “*a”* on the current state “*s”* when MDP follows policy *π*; *γ* is a discount factor with a value range of [0,1]. The reason for introducing a discount factor is that forward benefits have a certain degree of uncertainty and sometimes we prefer to obtain some rewards as soon as possible, so we need to make some deductions for forward benefits. *γ* values close to 1 focus more on long-term cumulative rewards, while *γ* values close to 0 consider short-term rewards. The details of MDP in this study can be found in Multimedia Appendix 1.

#### Computational Model

##### Overview

Patient states encompassed demographic information, vital signs, and laboratory results recorded at each time point. The actions involved adjustments to temperature and MAP. An RL agent, functioning as an AI clinician, made decisions based on the patient’s current state, determining the appropriate temperature and MAP control ranges. Following the implementation of these adjustments, patients transitioned to subsequent states, and the agent received reward feedback, guiding future decisions. To optimize cumulative rewards, we utilized the WD3QNE algorithm. WD3QNE develops a scoring system to assess recommended temperature and MAP ranges based on patient health states, enhancing decision quality through iterative score improvement. This algorithm is adept at managing sparse and delayed reward signals, making it particularly effective in addressing patient heterogeneity in treatment responses and delayed treatment efficacy indicators. Such a framework supports adaptive medical decision-making systems, accommodating a variety of patient scenarios, even in the presence of discontinuous reward signals or delayed clinical responses, thereby optimizing treatment strategies. The specifics of the states, actions, and rewards are delineated as given in the following sections.

##### State

The state space comprises consolidated patient clinical characteristics, with the SOFA score excluded from the state space but utilized as an intermediate reward during the training phase.

##### Action

Interventions for temperature and MAP are administered every 2 hours. A 6x6 action matrix is used, where temperature and MAP measurements at each time point are converted into integers corresponding to their respective sextiles, thereby defining the action space.

##### Reward

The primary focus of our reward system is patient survival, with rewards assessed after a sequence of clinical decisions. Additionally, we incorporate intermediate rewards, reflecting changes in the SOFA score, and final rewards based on survival status at 28 days.


$$r\;=\left\{ \begin{array}{l} \beta_{s}\times \left(SOFA_{t+1}-SOFA_{t}\right)\;\;t<T\\ R_{T}\;\;\;\;\;\;\;\;\;\;\;\;\;\;\;\;\;\;t=T \end{array}\right.\;$$

*R_T_* represents patient survival as 50 or death as −50. The reward parameter $\beta_{s}$ is set to −0.3.

##### Discount Factor

This parameter balances the consideration of future long-term rewards against immediate rewards in the RL model, with possible values ranging from 0 to 1. We selected a discount factor of 0.99, indicating that late-stage mortality is given nearly equal importance as early-stage mortality in the decision-making process.

### Clinician and Agent Policy Construct

Clinician policy is defined as the continuous clinical decision-making trajectory reconstructed based on real-world electronic health record data, with its core features focusing on the management of target intervals for body temperature and MAP, presenting the following modeling characteristics:

1. Modeling of the action space: the temperature and MAP regulation strategies are discretized into a 6×6 gridded action space.
2. Temporal decision dynamics: with a decision interval unit of 2 hours, the state-action pair mapping relationship is constructed through the real-time temperature, MAP target values, and corresponding physiological indicators (such as heart rate and lactate level) recorded in the electronic health record.
3. Integration mechanism of background interventions: explicit exclusions-other treatment measures are not included in the RL action space. Implicit coupling paths-the physiological effects of background interventions are dynamically reflected through multi-dimensional state vectors (such as white blood cell count), and the cumulative impact of background treatments on organ functions is systematically captured through the design of SOFA score and the end-point mortality in the composite reward function.

AI policy is a bivariate joint optimization strategy for temperature and MAP generated through deep RL, featuring the following:

1. Policy consistency: strictly reusing the 6×6 discrete action space of clinician policy.
2. Differentiated optimization objectives: maximizing the reward function based on survival end points and dynamic changes in SOFA by regulating the temperature-MAP combination.
3. Boundaries of clinical adaptability: first, the AI strategy does not change the existing treatment framework but only provides dynamic adjustment suggestions for the management of temperature and MAP. Second, other treatment measures, as uncontrollable covariates, have their effects continuously transmitted to the next decision cycle through the Markovian assumption of state observables. Third, the final conclusion regarding survival benefits is strictly limited to the additional benefits obtained through refined management of body temperature and blood pressure within the context of current standard treatments.

### Off-Policy Evaluation

In model evaluation, the effectiveness of the AI-derived policy is assessed by comparing it to health state trajectories generated by human clinicians. We use a doubly robust off-policy value evaluation method, which integrates importance sampling with an approximation of the MDP, to compute unbiased estimates for each trajectory. The calculation formula is as follows:

Where *ρ* represents the importance ratio between the AI policy π_1_ and the clinician policy $\pi_{0}\;:\;\rho =\frac{\pi_{1}}{\pi_{0}}$. 𝑉̂ (𝑆_𝑡_) is the evaluation value. $\hat{Q}(S_{t},a_{t})$ is the expected return of taking action *a* under state *S_t_*.

**Figure 1.**
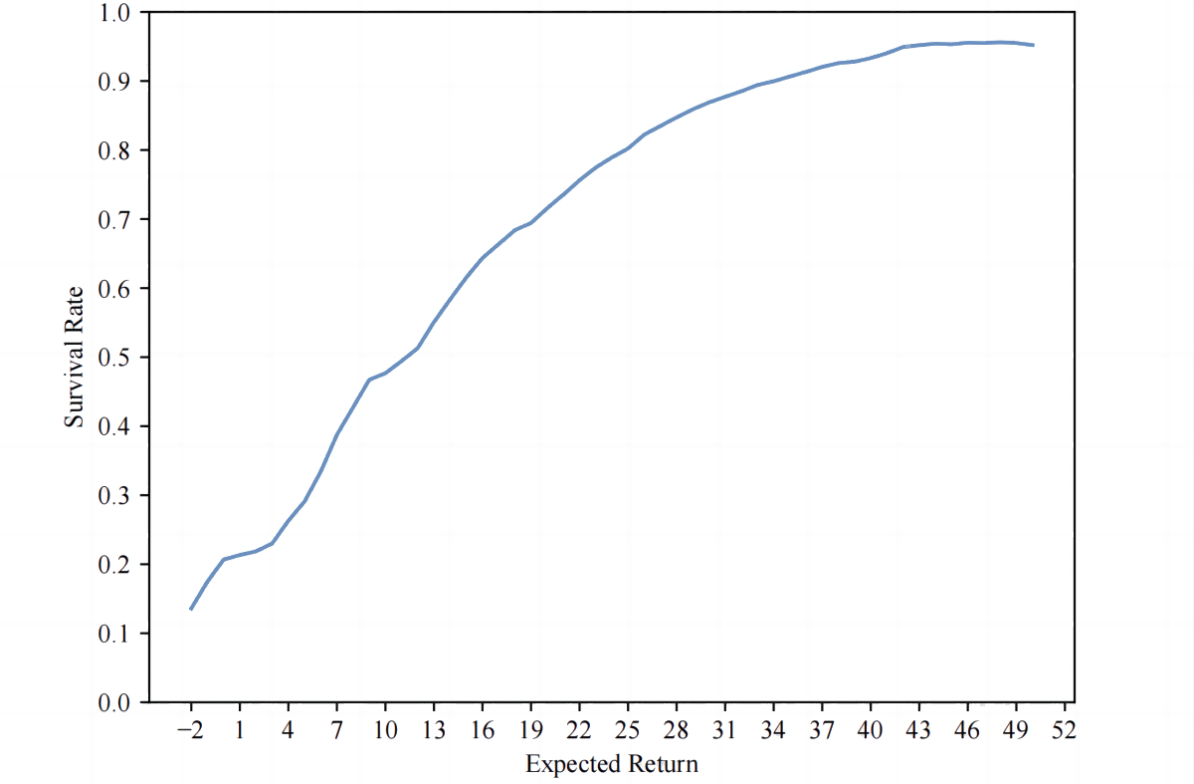
The relationship between expected return and survival rate.

To further evaluate the survival rate of the policy, we applied a policy-based State-Action-Reward-State-Action RL algorithm to establish the relationship between expected return and survival rate: $Q(S_{t},a_{t})\leftarrow Q(S_{t},a_{t})+\alpha \left(r+\gamma Q(S_{t+1},a_{t+1})-Q(S_{t},a_{t})\right)$. First, the expected return value *V* is computed. Then, based on the return value, we calculate the average survival rate. The survival formula is as follows:


$$S\left(Q_{i}\right)=\frac{sur_{V_{i}}}{tal_{V_{i}}}$$

where ${sur}_{V_{i}}$ represents the number of survivors, ${tal}_{V_{i}}$ represents the total population given the expected return *V_i_. V_i_* is an integer and $V_{t}\in V_{i}$. The relationship between expected return and survival rate is illustrated in Figure 1. The survival rate is positively correlated with the expected return.

## Results

### Model Performance

**Figure 2.**
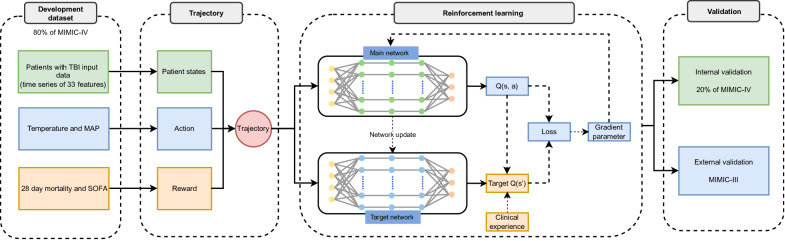
Flowchart of analysis. MAP: mean arterial pressure; MIMIC: Medical Information Mart for Intensive Care; SOFA: Sequential Organ Failure Assessment; TBI: traumatic brain injury.

The data analysis process is illustrated in Figure 2. We included a total of 2745 patients from the MIMIC-IV database, who were randomly allocated into training (n=2198) and internal validation (n=547) cohorts in an 8:2 ratio. For external validation, 2463 patients with TBI were sourced from the MIMIC-III database. The clinical characteristics of the study population are detailed in Table 1.

**Table 1.** Baseline characteristics of the study population.

	Train (n=2198)	Internal validation (n=547)	External validation (n=2463)
Age (years), mean (SD)	62.7 (22.2)	63.0 (21.6)	75.1 (67.3)
Sex (male), n (%)	1362 (62.0)	343 (62.7)	1518 (61.6)
Length of hospital stay (days), mean (SD)	9.12 (10.8)	9.40 (12.4)	9.42 (11.0)
Length of ICU^a^ stay (days), mean (SD)	3.84 (5.49)	4.09 (5.53)	4.37 (5.84)
Hospital mortality, n (%)	284 (12.9)	68 (12.4)	395 (16.0)
28-day mortality, n (%)	362 (16.5)	92 (16.8)	446 (18.1)

a ICU: intensive care unit.

**Figure 3.**
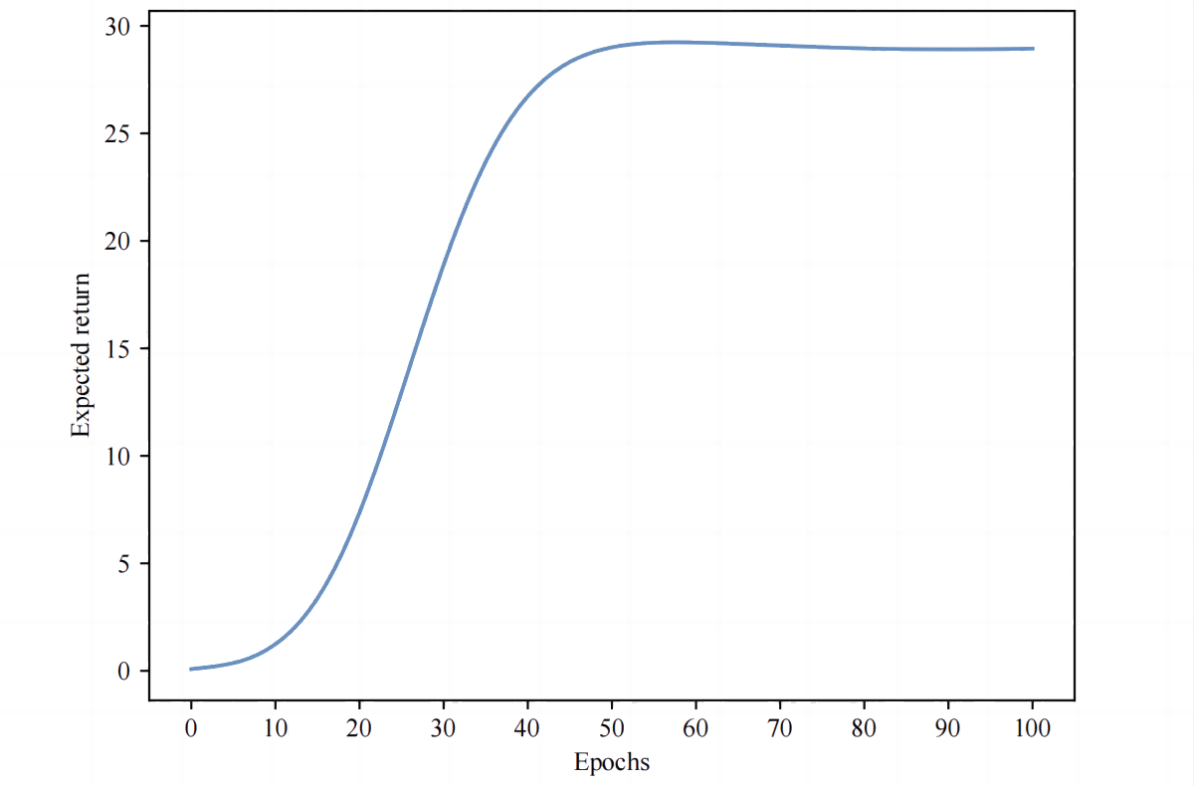
Expected return of algorithm at each learning epoch.

Initially, we used the MIMIC-IV dataset to estimate the expected return using a doubly robust off-policy evaluation method. Our findings reveal that the survival rate under the AI policy surpasses that under the clinician policy. Specifically, the survival rate with the AI policy is 88.016% (95% CI 85.191%‐90.840%), with an expected return of 28.978 (95% CI 28.797‐29.160). In contrast, the survival rate under the clinician policy is 81.094% (95% CI 80.422%‐81.765%), with an expected return of 27.092 (95% CI 24.584‐29.600). Furthermore, Figure 3 illustrates the expected return across epochs, showing convergence and stabilization around a reward value of 29.

### Model Strategy

**Figure 4.**
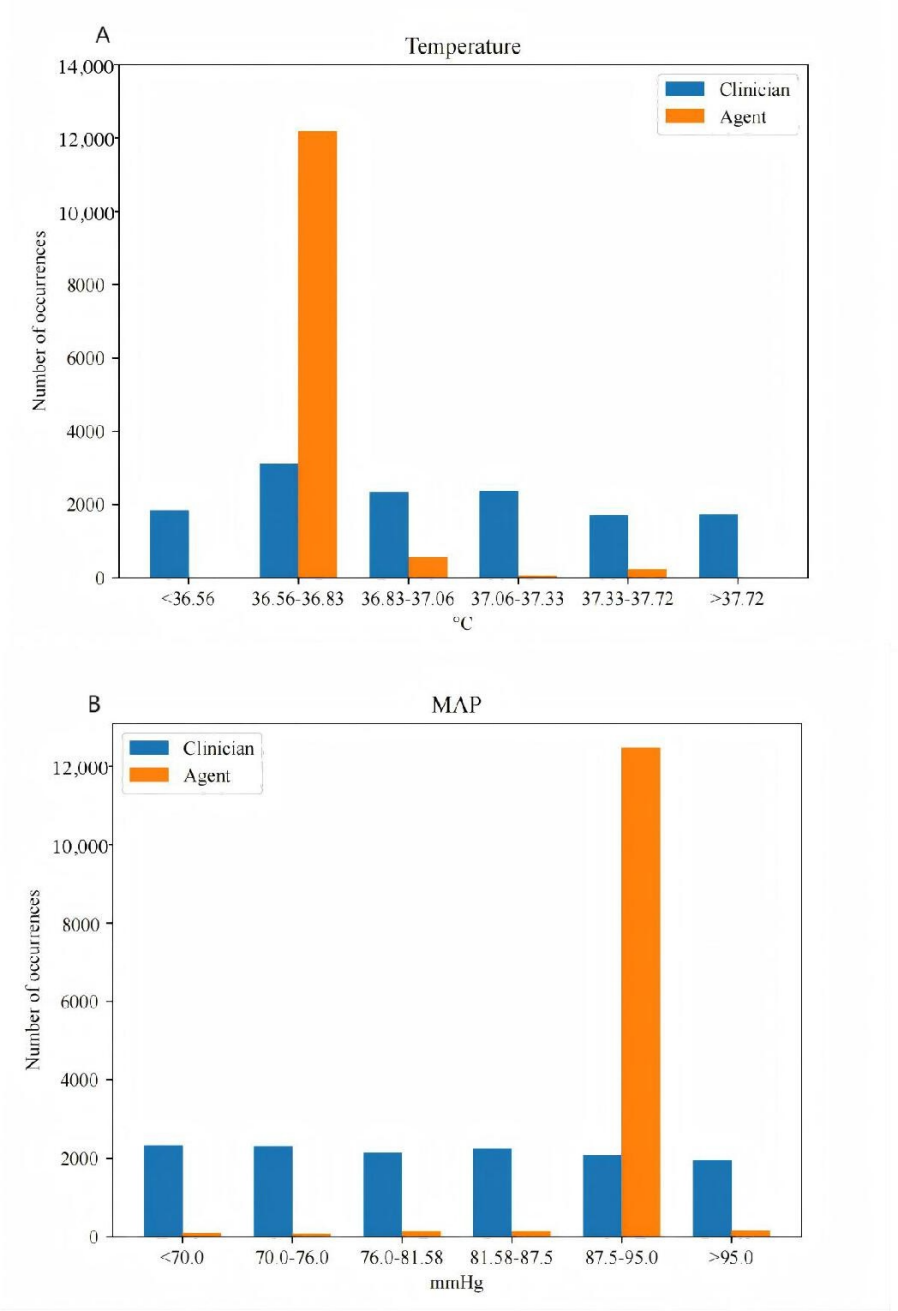
Visualization of the action distribution. The test set includes 13,075 decision time instances and the designed model facilitates 16 action bins in the action space. (A) Temperature (℃) and (B) MAP (mm Hg). MAP: mean arterial pressure.

The frequency distribution of the optimal AI strategy was compared with that of clinicians, with detailed analyses conducted for temperature and MAP adjustments, as presented in Tables2  3, and Figures4  5. The AI algorithm more frequently selected normal temperature ranges (36.56 °C to 36.83 °C), with an increase of 291.52% compared with clinicians, while selecting fewer temperatures outside the range of<36.56 °C and >37.72 °C. Additionally, the AI recommended MAP levels of 87.5‐95.0 mm Hg, 498.46% more often than clinicians.

**Table 2.** Distribution of the chosen action by artificial intelligence in comparison to the clinicians’ performance.

	Range 1	Range 2	Range 3	Range 4	Range 5	Range 6
Temperature (℃)	<36.56	36.56‐36.83	36.83‐37.06	37.06‐37.33	37.33‐37.72	>37.72
Difference, n (%)	−1837 (−14.05)	9075 (69.41)	−1753 (−13.41)	−2299 (−17.58)	−1465 (−11.2)	−1721 (−13.16)
MAP^a^ (mm Hg)	<70.0	70.0‐76.0	76.0‐81.58	81.58‐87.5	87.5‐95.0	>95.0
Difference, n (%)	−2242 (−17.15)	−2230 (−17.06)	−2006 (−15.34)	−2109 (−16.13)	10,383 (79.41)	−1796 (−13.74)

a MAP: mean arterial pressure.

**Table 3.** Comparison of percentage of change for each action bin between artificial intelligence policy and clinician policy.

	Range 1	Range 2	Range 3	Range 4	Range 5	Range 6
Temperature (℃)	<36.56	36.56‐36.83	36.83‐37.06	37.06‐37.33	37.33‐37.72	>37.72
Difference, n (%)	–1837 (−100)	9075 (291.52)	–1753 (−75.07)	–2299 (−97.21)	–1465 (−85.97)	– 1721 (−100)
MAP^a^ (mm Hg)	<70.0	70.0‐76.0	76.0‐81.58	81.58‐87.5	87.5‐95.0	>95.0
Difference, n (%)	–2242 (−96.35)	–2230 (−96.96)	–2006 (−93.22)	–2109 (−93.4)	10,383 (498.46)	–1796 (−91.87)

a MAP: mean arterial pressure.

**Figure 5.**
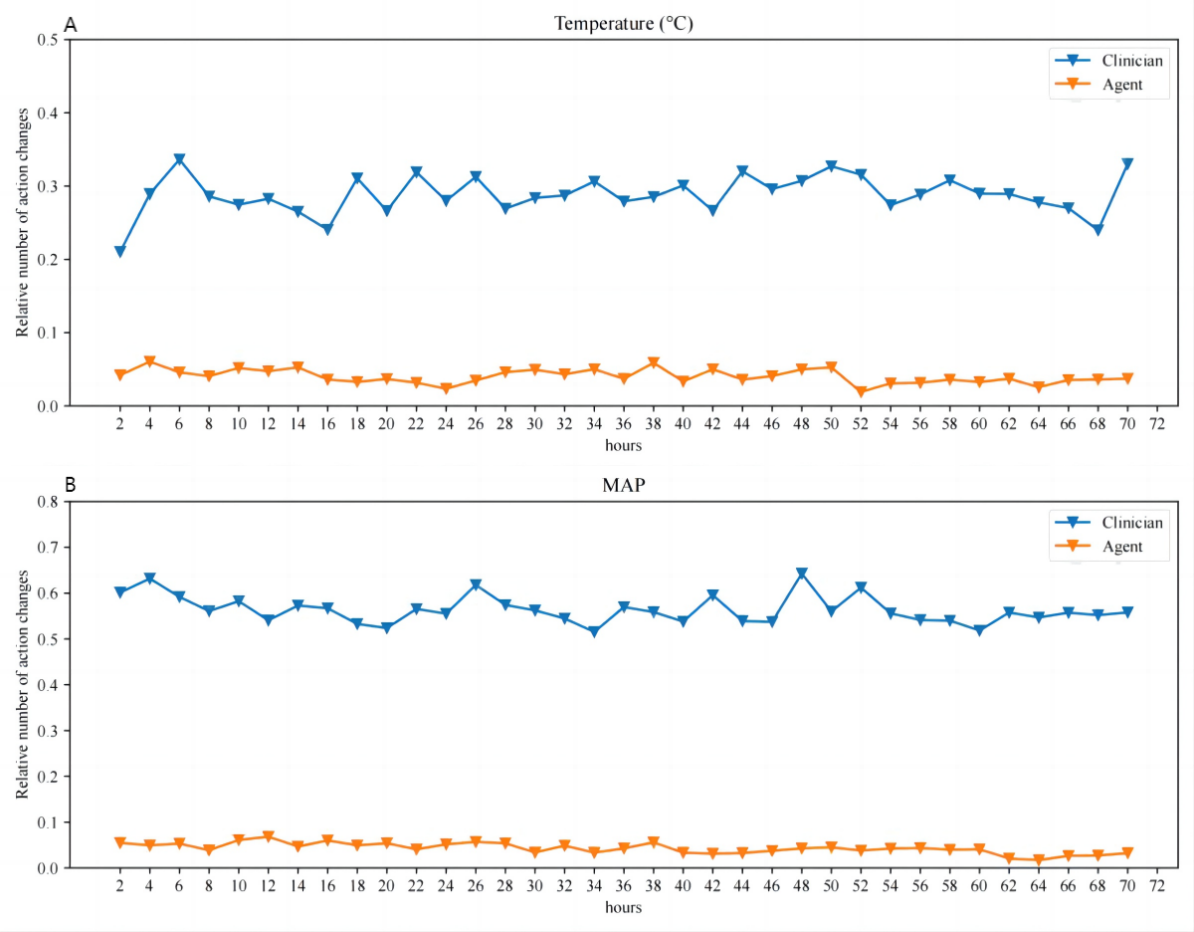
Number of action changes. The relative number of action changes (temperature and MAP) is shown in relation to the number of patients with traumatic brain injury at each 2-hour time step. Clinicians’ action changes are shown in blue while the artificial intelligence action changes are shown in orange. (A) Temperature (℃) and (B) MAP (mm Hg). MAP: mean arterial pressure

Further analysis of the number of action changes made by the AI within each 2-hour time step over a 72-hour period revealed a consistently lower frequency of adjustments compared with clinicians (Figure 4), indicating the AI algorithm’s preference for stable vital sign management. Feature importance for temperature and MAP changes was assessed using an out-of-bag analysis with random forests (Table 4). The top 5 clinical features influencing optimal temperature and MAP selections were age, heart rate, pO2, hemoglobin, and lactate. The importance weights for temperature and MAP adjustments were found to be equal.

**Table 4.** Out-of-bag feature weight analysis of artificial intelligence. The relative weight of each feature using out-of-bag feature weight analysis, based on the relative loss of prediction, represented by an increase of the mean squared error.

Variables and features	Importance
Temperature (℃)	
Age (years)	0.072
Heart rate	0.068
SIRS^a^	0.062
WBC^b^	0.045
Respiratory rate	0.040
PH^c^	0.037
aPTT^d^	0.035
PO2	0.034
Calcium	0.034
Magnesium	0.033
Platelet	0.031
PT^e^	0.030
SpO2	0.030
Glucose	0.029
Lactate	0.029
PCO2	0.028
Chloride	0.027
Bicarbonate	0.027
Creatinine	0.024
Bilirubin total	0.024
Hemoglobin	0.024
PaO2/FiO2	0.023
FiO2	0.023
Potassium	0.023
Sodium	0.023
AST^f^	0.023
Urine output	0.022
INR^g^	0.021
Base excess	0.021
Bands	0.018
BUN^h^	0.017
GCS^i^	0.013
ALT^j^	0.010
MAP^k^ (mm Hg)	
Age (years)	0.068
Heart rate	0.066
PO2	0.051
Hemoglobin	0.050
Lactate	0.043
Urine output	0.035
Platelet	0.034
AST	0.034
SpO2	0.033
aPTT	0.033
Calcium	0.033
WBC	0.033
Magnesium	0.032
Respiratory rate	0.032
Creatinine	0.031
Glucose	0.031
PCO2	0.029
PT	0.029
INR	0.029
PH	0.029
PaO2/FiO2	0.028
FiO2	0.027
Bilirubin total	0.027
Chloride	0.024
Base excess	0.023
Potassium	0.021
Bicarbonate	0.021
Sodium	0.020
BUN	0.019
Bands	0.017
GCS	0.010
ALT	0.008
SIRS	0.005

a SIRS: Systemic Inflammatory Response Syndrome.

b WBC: white blood cell.

c PH: potential of Hydrogen.

d aPTT: activated partial thromboplastin time.

e PT: prothrombin time.

f AST: aspartate aminotransferase.

g INR: international normalized ratio.

h BUN: blood urea nitrogen.

i GCS: Glasgow Coma Scale.

j ALT: alanine aminotransferase.

k MAP: mean arterial pressure.

### External Validation

External validation was conducted using data from the MIMIC-III database, which included a total of 2463 patients with TBI. The results show that the survival rate under the AI policy is 87.565% (95% CI 86.158%‐88.972%), with an expected return of 27.517 (95% CI 27.603‐27.431).

## Discussion

### Principal Findings

We used the WD3QNE algorithm to develop an optimal 28-day survival strategy for patients in the ICU with TBI. This AI algorithm prioritizes the management of MAP and temperature to derive the optimal value function. The AI algorithm demonstrated improved 28-day survival rates in both the internal and external validation datasets.

The WD3QNE algorithm builds upon the foundations of the double deep Q-networks with dueling networks and dueling deep Q-network RL algorithms by incorporating a target Q-value function with adaptive dynamic weights, enhancing estimation accuracy, accelerating convergence, and improving stability. By integrating clinical expertise, the WD3QNE algorithm further enhances decision-making performance (Multimedia Appendix 2). In this AI model, intermediate rewards are implemented to expedite the ML process by providing more frequent feedback. The SOFA score was selected for intermediate rewards due to its strong association with patient severity and mortality rates in the ICU [16,17]. Unlike lactate, lactate clearance rate, and base excess, the SOFA score uniquely differentiates between survival and death at the time of admission [18].

In this study, the AI algorithm prioritizes specific temperature (36.56 °C to 36.83 °C) and MAP (87.5‐95.0 mm Hg) action intervals, resulting in improved outcomes and higher survival rates. Effective TBI management focuses on preventing hypotension, and hypoxia, and maintaining appropriate cerebral perfusion to mitigate secondary brain injury [4]. MAP—an easily obtainable and routinely monitored parameter—was used as a key indicator. Compared with systolic blood pressure (SBP), MAP better reflects cerebral perfusion (CPP; CPP=MAP–ICP). In pediatric TBI, lower MAP has been found to predict adverse outcomes (AUC=0.75) [19]. Lower MAP and high SBP variability are associated with increased mortality in brain injury patients [20]. While fewer clinical studies focus on MAP, research on SBP suggests maintaining SBP above critical thresholds is beneficial. Recent guidelines recommend keeping SBP above 100 mm Hg for patients with TBI aged 50‐69 years, and above 110 mm Hg for those aged 15‐49 years or over 70 years [21-23]. These findings align with our AI algorithm’s preference for a higher MAP range of 87.5‐95.0 mm Hg, which may benefit patients with TBI by ensuring better cerebral perfusion. Changes in MAP influence cerebral blood vessel constriction and dilation, thereby affecting ICP. Increasing MAP can reduce ICP and help control intracranial pressure [24]. In severe TBI cases where the autoregulatory function is lost, ICP trends consistently with MAP changes [25]. Thus, hypotension below the range of cerebral vascular autoregulation leads to hypoperfusion, secondary cerebral ischemia, and hypoxia. Elevating blood pressure within the autoregulatory range, or in patients with TBI with impaired autoregulation, can optimize cerebral blood flow under cerebral blood flow monitoring. This underscores the importance of adjusting pressure to improve cerebral blood flow and suggests further exploration into optimizing oxygen metabolism in patients with TBI. Patients with TBI are subject to a cascade of pathological alterations, including secondary brain edema, mitochondrial dysfunction, calcium overload, and inflammatory responses [26].

In the ICU, in addition to analgesia, sedation, and mechanical ventilation to balance oxygen supply and demand, temperature management remains a critical component. Preclinical studies have demonstrated that hypothermia can substantially reduce neuronal cell death and mitigate brain ischemia-reperfusion injury [27-29]. However, clinical trial outcomes regarding hypothermia’s impact on the prognosis of patients with brain injury have been inconsistent [30-34]. These inconsistencies arise from significant variations in patient populations, temperature ranges, durations of hypothermia, and follow-up periods, underscoring the need for more rigorous research [35]. The latest fourth edition guidelines for TBI specify that early (within 2.5 h postinjury), short-term (within 48 h postinjury) prophylactic hypothermia is not recommended to enhance outcomes in patients with diffuse brain injury [36]. In contrast to the ongoing debate regarding hypothermia (<36 °C) treatment for patients with TBI, fever (>38 °C) is recognized as an adverse prognostic factor affecting TBI outcomes [37]. Most of the patients with TBI experience fever, which can lead to increased intracranial pressure, cerebral metabolism, and exacerbation of ischemic injury. The study by Birg et al [38] confirmed that when brain temperature exceeds 37.5 °C, intracranial pressure increases, resulting in decreased cerebral perfusion pressure‌. The investigation by Puccio et al [39] further validated that intravascular cooling can ameliorate intracranial hypertension and mitigate secondary injuries. Consequently, the Seattle International Severe Traumatic Brain Injury Consensus Conference guidelines advocate for fever control in patients with TBI. In instances where primary and secondary interventions for intracranial hypertension prove ineffective, hypothermia treatment (35 °C‐36 °C) may be considered [40]. In 2024, an expert consensus on TBI temperature management was specifically proposed. Experts believe that controlling the body temperature within 36.0 °C‐37.5 °C is the basis for managing patients with TBI [41]. In the present study, the AI algorithm exhibits a propensity towards the lower temperature range of 36.5 °C‐36.8 °C. Compared with both lower and higher temperature ranges, this interval mitigates the risk of arrhythmias, compromised circulation, and coagulation abnormalities induced by hypothermia, without exacerbating intracranial pressure and cerebral oxygen metabolism disturbances. Finally, results from the out-of-bag analysis underscore age, heart rate, pO2, hemoglobin, and lactate as the primary state variables influencing the AI’s selection of optimal MAP and temperature.

TBI is a complex condition and it is likely that no single factor entirely accounts for the disease outcome. Currently, there is active exploration of novel methods to enhance TBI monitoring, diagnosis, and assessment, particularly through the identification of new biomarkers for brain injury [42]. In the field of neurocritical care, clinicians have begun to use multimodal monitoring approaches, utilizing various invasive or noninvasive methods. Treatment strategies are adjusted by measuring distinct cerebral physiological parameters (primarily cerebral blood flow, metabolism, and oxygenation), with temperature and blood pressure emerging as the most critical and readily observable influencing factors. A 2021 meta-analysis of multiple TBI management guidelines revealed that guideline implementation correlates with improved prognoses [43]. However, as evidence grading standards have become increasingly stringent, the number of strong recommendations has progressively diminished, resulting in reduced clinical decision-making support [44]. While there remains no unified opinion regarding therapeutic hypothermia for patients with cerebral herniation or severe intracranial hypertension, even when implementing hypothermia protocols, experts recommend targeting near-normal temperature ranges. Blood pressure management involves multiple variables including vascular volume, central venous pressure, and vascular tone, with individual therapeutic responses significantly impacting cerebral perfusion [45]. Consequently, the urgent need for precision medicine in TBI treatment has become particularly evident.

RL emphasizes exploration and exploitation, and its core is the dynamic change of strategies and values. In this study, we adopt the dynamical weight of the behavioral differences between the clinician strategy and the AI strategy through the importance ratio (ρ). The core idea is to jointly offset the confounding effects through inverse probability weighting (IPW) and the outcome model. In addition, we also adopt a SOFA-stratified human-machine collaborative strategy. For mild patients with SOFA <5, the historical decision-making data of clinicians are directly used as the Q-value function. This is equivalent to decoupling the effect of real-world doctor interventions (do-action) from the evolution of potential complications, avoiding new confounders introduced by AI due to wrong interventions in low-risk scenarios. For severe patients with SOFA ≥5, we completely rely on the RL strategy. Here, it is assumed that the dynamic changes of complications play a stronger leading role in the evolution of the endogenous state of the human body than the intervention effect, so AI is allowed to explore freely. In addition, the state vector of our MDP includes dynamic physiological indicators (such as lactate level) closely related to complications, and these indicators can be used as proxy variables for complications to reduce unobserved confounders. For example, lactate has been proven to be an early warning indicator of shock and is continuously tracked in the state-update cycle (once every 2 h).

It should be noted that the MDP model cannot completely simulate the real world. To some extent, the MDP model is a simplified version of the real world. There may be the following deviations between the 2: for instance, the MDP assumes perfect observation and modeling of state transitions following medical interventions, whereas real-world scenarios involve measurement errors and undocumented latent factors (eg, genetic variations, pharmacokinetic differences). Additionally, the model presumes flawless execution of therapeutic actions, while practical implementations may encounter instrumentation inaccuracies. To mitigate these discrepancies, the doubly robust evaluation corrects 2 sources of errors through a hybrid mechanism of IPW and adjustment of model fitting residuals. First of all, IPW is used to quantify the probability ratio of AI strategy actions to doctor strategy actions (to address the difference in action frequencies of different strategies); residual compensation uses the real survival status of patients to correct prediction errors (to address the inherent bias of the MDP state transition model). For example, for the historical record of patient A, if the doctor actually used “low temperature” (and the patient survived), while the AI suggested “high temperature” (with an increased predicted survival probability in the MDP), we would calculate the importance weight of the “high temperature” action (based on the difference between the AI and doctor strategies); and the adjusted survival rate estimate (the 88% survival rate has been corrected by this method, rather than being the direct output of the original MDP). Constrained by ethical and privacy regulations, we are temporarily unable to directly apply AI strategies to real patients. In the next step of our plan, we are going to carry out “AI-assisted decision-making” (only for doctors’ reference) among a small number of critically ill patients to gradually accumulate actual efficacy data.

The management of patients with TBI in the ICU is a continuous and dynamic process. Clinicians in the ICU are inundated with extensive clinical data, necessitating timely and rational decision-making, a process that poses significant challenges. In contrast, RL algorithms dynamically adjust actions, such as MAP and temperature, by interacting with the environment to maximize cumulative rewards. By integrating clinical expertise and adaptive weights into the Q-value function, our algorithm enhances performance and optimizes the 28-day survival rate of patients, demonstrating clear practicality and applicability in clinical settings, particularly in the absence of multimodal monitoring. However, this study has several limitations. First, our sample is derived solely from the MIMIC database in the United States, lacking external validation from other regions. Second, the AI strategy focuses exclusively on key actions related to MAP and temperature, omitting other critical variables such as trauma factors, surgical interventions, and ventilator settings. Third, RL agents must learn from limited data and intervention variations collected offline. Using trial and error to explore all possible situations may conflict with medical ethics, limiting the ability of RL agents to try new behaviors to discover those with higher rewards and better long-term outcomes [11]. As a result, this AI strategy may not represent the optimal solution for reducing TBI mortality rates. Further research is required to incorporate and validate additional relevant variables and parameters to refine and improve the AI strategy.

To mitigate potential overfitting in the RL model, we implemented several measures during the study. For instance, continuous variables were normalized, and an external validation dataset (MIMIC-III) was used to enhance and test the model’s generalization ability. However, we acknowledge that there may still be residual overfitting risks. In future research, we will further optimize the model. On one hand, we plan to collect data from more diverse sources and types, including patients from different medical institutions and regions, to enrich the diversity of training data and mitigate the impact of data bias on the model. On the other hand, we will explore ways to improve the model architecture, such as incorporating more advanced regularization techniques to prevent the agent from over-relying on specific patterns in the training data and enhance its adaptability to new data and complex clinical scenarios. Additionally, we will conduct in-depth analyses of the RL agent’s behavior across patients with different characteristics, performing sensitivity analyses to gain a deeper understanding of the model’s decision-making process. This approach will help identify potential overfitting risks and guide targeted improvements. By addressing these aspects, we aim to develop a more robust and generalizable model for clinical decision support. The main algorithm of this study has been uploaded to Multimedia Appendix 3.

### Conclusions

In summary, we used a novel RL algorithm to enhance the 28-day survival rate of patients with TBI in the ICU. This algorithm showed superior performance across the training set, validation set, and external validation, with AI-driven decision-making resulting in higher survival rates compared with clinician-directed care. This RL algorithm for patients with TBI indicates that a more personalized and targeted optimization of the vital signs is possible. It will assist clinicians in making decisions on an individualized patient-by-patient basis.

## Supplementary material

10.2196/63847 Multimedia Appendix 1 Markov decision process description.

10.2196/63847 Multimedia Appendix 2 WD3QNE algorithm description. WD3QNE: weighted dueling double deep Q-network with embedded human expertise.

10.2196/63847 Multimedia Appendix 3 TBI_algorithm code. TBI: traumatic brain injury.

## Abbreviations

AI: artificial intelligence
ICP: intracranial pressure
ICU: intensive care unit
IPW: inverse probability weighting
MAP: mean arterial pressure
MDP: Markov decision process
MIMIC: Medical Information Mart for Intensive Care
ML: machine learning
RL: reinforcement learning
SOFA: Sequential Organ Failure Assessment
TBI: traumatic brain injury
WD3QNE: weighted dueling double deep Q-network with embedded human expertise
