# Supplementary material for: Optimizing Vital Signs in Patients With Traumatic Brain Injury: Reinforcement Learning Algorithm Development and Validation
Source: J Med Internet Res. 2025 Jul 3;27:e63847. doi: 10.2196/63847 (PMC12244269; doi:10.2196/63847)
Supplement: Multimedia Appendix 1 [file jmir-v27-e63847-s001.docx]

****Markov Decision Process (MDP)****

Markov Decision Process

We simulated the health trajectories and clinical decisions of ICU patients using a Markov Decision Process (MDP), which is a continuous interaction process between the agent and the environment. By defining five elements: state space (***S***), action space (***A***), state transition probability (***P***), reward function (***R***) and discount factor (***γ***), the interaction process between the agent and the environment is transformed into a computable model. In 2010, Alagoz et al. proposed using MDP to solve the problem of sequential clinical treatment under uncertainty, and subsequently more researchers have focused on clinical decision analysis in reinforcement learning.

The specific analysis process is as follows: the intelligent agent selects action at based on the current state ***S_t_***; For state ***S_t_*** and action ***A_t_***, MDP obtains ***R_t_*** and ***S_t+1_*** based on the reward function and state transition function, and feeds them back to the agent. The goal of an intelligent agent is to maximize the accumulated reward obtained. The function by which an intelligent agent selects an action from the set of actions ***A*** based on its current state is called strategy ***π***. The strategy ***π(a|s)=P(A_t_=a|S_t_=s)*** is a function that represents the probability of taking action ***a*** after the input state ***s***. When a strategy is a stochastic policy, it outputs a probability distribution of actions each state, and then samples based on this distribution to obtain an action. In MDP, the state value function ***V^π^(s)*** based on policy ***π*** is the expected return that can be obtained by following policy ***π*** starting from state ***s***; In addition, due to the existence of actions, an additional action value function ***Q^π^*** ***(s,a)*** based on policy ***π*** is defined to represent the expected return obtained by performing action ***a*** on the current state ***s*** when MDP follows policy ***π***; ***γ*** is a discount factor with a value range of [0,1]. The reason for introducing a discount factor is that forward benefits have a certain degree of uncertainty and sometimes we prefer to obtain some rewards as soon as possible, so we need to make some deductions for forward benefits. ***γ*** values close to 1 focus more on long-term cumulative rewards, while ***γ*** values close to 0 consider short-term rewards. This study are specifically designed as follows:

1. **State Space**

The patient's clinical status is dynamically composed of multidimensional physiological indicators and laboratory parameters, these features are mainly divided into the following three categories:

**①Static features:** age.

**②Dynamic physiological indicators:** Heart rate, respiratory rate, temperature, MAP, FiO2, PO2, PaO2/FiO2, PCO2, SpO2, urineoutput, GCS, SIRS.

**③Laboratory parameters:** Lactate, glucose, BUN, creatinine, WBC, platelets, PT, aPTT, INR, pH, bicarbonate, sodium, potassium, calcium, magnesium, chloride, bilirubin total, hemoglobin, AST, ALT, baseexcess, bands.

**Data processing and time window:**

**Time resolution:** Collect data from the first 72 hours after ICU admission, with a 2-hour time step (a total of 36 time steps).

**Missing value handling:** Forward filling is performed on continuous measurement values, and the remaining missing values are interpolated using the k-nearest neighbor algorithm (KNN, k=3).

**Normalization:** Continuous variables are mapped to the [-1,1] interval through Min-Max normalization.

**Special treatment:** Sequential Organ Failure Assessment (SOFA) as an intermediate reward signal, not included in the state space.

1. **Action Space**

Every 2 hours, temperature and MAP are jointly regulated to form a 6 × 6 discrete action grid. The specific division is based on the following table. The measured values at each time step are mapped to the corresponding sixth percentile interval, forming 36 possible joint regulation strategies

|  | 1 | 2 | 3 | 4 | 5 | 6 |
| --- | --- | --- | --- | --- | --- | --- |
| **Temperature (℃)** | **<36.56** | **36.56-36.83** | **36.83-37.06** | **37.06-37.33** | **37.33-37.72** | **>37.72** |
| **MAP (mmHg)** | **<70.0** | **70.0-76.0** | **76.0-81.58** | **81.58-87.5** | **87.5-95.0** | **>95.0** |

1. **Reward Function**

The reward function design takes into account both short-term physiological improvement and long-term survival goals, and the formula is as follows:

$$r =\left\{ \begin{aligned} \beta_{s}\times({SOFA}_{t+1}-{SOFA}_{t}) t<T \\ R_{T} t=T \end{aligned} \right.$$

****Intermediate Reward:****

${SOFA}_{t+1}-{SOFA}_{t}$

**Reflect dynamic changes in organ function,β_s_=-0.3, which means a reward of+0.3 for every 1 point decrease in SOFA, encouraging the reversal of organ failure trend.**

****Terminal Reward:****

$R_{T}$=+50（28 day survival）or $R_{T}$=−50（28 day death）

1. **Discount factor (γ):**

Set to 0.99, emphasizing the importance of long-term survival benefits and avoiding strategies overly biased towards short-term SOFA improvement.

1. **State transitions**

The state transition probability is not explicitly modeled, and the dynamic environment is implicitly learned from the patient's real trajectory data. The next state s_t+1_ is determined by the current state s_t_, action a_t_ and the patient's physiological response.
