# Supplementary material for: Optimizing Vital Signs in Patients With Traumatic Brain Injury: Reinforcement Learning Algorithm Development and Validation
Source: J Med Internet Res. 2025 Jul 3;27:e63847. doi: 10.2196/63847 (PMC12244269; doi:10.2196/63847)
Supplement: Multimedia Appendix 2 [file jmir-v27-e63847-s002.docx]

Supplementary File

Traditional machine learning relies on manually designed features and feature extraction, while reinforcement learning finds a policy that maximizes cumulative rewards through interaction with the environment. The learning process is based on trial and feedback. Data is generated in real-time by the agent interacting with the environment, focusing on sequential decision problems. The agent needs to take actions based on the current state of the environment and consider the impact of current actions on future rewards.

**Q-learning**

1. learning is a classic reinforcement learning algorithm used to solve control problems in Markov Decision Processes[1]. It is based on the concept of value iteration, where the agent estimates the Q-value, a value function for each state-action pair, to guide its selection of the optimal action in each state. Q-learning is a value-based algorithm, where Q refers to $Q(s,a)$, which represents the value of taking action $a$ in state $s$. The main idea of this algorithm is to construct a Q-table to store Q-values by mapping states and actions, and then select the action that yields the maximum reward based on the Q-values.

**Deep Q-Networks (DQN)**

When Q-learning is applied to more complex tasks, the tabular representation of the Q-value function becomes impractical due to the large state space, making it infeasible to store a Q-value for every state-action pair. Therefore, DQN approximate the Q-value function using deep neural networks, enabling the algorithm to handle high-dimensional inputs[2,3]；We can approximate the action-value function $Q(s,a)$ using a function $Q(s,a;\omega)$, which is referred to as value function approximation, where $\omega$ represents the parameters learned through the neural network training process. Neural network training uses the difference between the network's output and the target values as the loss function. Through gradient descent method is employed to update the neural network parameters, with the goal of minimizing the loss function. The target value for the Q-network is represented as:

$y_{t}=r_{t}+\gamma\max_{a} Q(s_{t+1},a;\omega)$;

Here, part of is derived from the Q-network’s own estimations and using to update the Q-network can lead to overestimation of Q-values. To mitigate this overestimation, the use of a target network has been proposed [4]. During the update process, only the weights $\omega$ of the evaluation network $Q(s,a;\omega)$ are updated. After a certain number of updates, the updated weights are copied to the target network $\omega-$. Since the target value remains relatively fixed during the period when the target network is not updated, the introduction of the target network helps increase the stability of the learning process.

**Double DQN**

Double DQN is designed to better address the overestimation problem by using the original Q-network $Q(s,a;\omega)$ to select the action $a*$that maximizes the Q-value, and then using the target network to compute the target value as follows:

$y_{t}=r_{t}+\gamma\max_{a} Q(s_{t+1},a^{*};\omega^{-})$;

This approach helps reduce the overestimation bias that arises from using the same network to both select and evaluate actions[5].

**Double DQN with Dueling Networks (D3QN)**

In Dueling Networks, the Q-network is split into two output values: the state-value function $V(s)$ and the advantage function $A(s,a)$. These two components are then combined to compute the Q-value:

$$Q(s,a)=V(s)+A(s,a)$$

The advantage of this structure is that the network does not need to learn the Q-values for every possible action at each state, thus making the learning process more efficient. The authors of the Dueling Network architecture recommend a specific method to combine these two functions[6]. By combining Double DQN with Dueling Networks, it is possible to reduce overestimation, though Double DQN may still suffer from underestimation issues[7].

**Weighted Dueling Double Deep Q-Network with embedded human Expertise (WDQ3NE):**

It features a target Q-value function with adaptive dynamic weights to improve estimation accuracy and integrates clinical expertise to enhance the performance of the reinforcement learning algorithm in decision-making.

The target Q-value function first incorporates an adaptive dynamic weight *p*:

$$Q(S_{t+1},a_{t+1}) = p\times\max_{a_{t+1}} Q(S_{t+1},a_{t+1};\omega^{-})+(1-p)\times Q(S_{t+1},arg\max_{a_{t+1}} Q(S_{t+1},a_{t+1};\omega){;\omega}^{-})$$

Where *ω* represents the parameters of the primary network, and *ω*^−^ represents the parameters of the target network. The calculation of the adaptive dynamic weight *p* is as follows:

Here, *p* is calculated by dividing the maximum target Q-value by the sum of the target Q-values across all possible actions. The target Q-values are obtained using the Dueling DQN:

$$\varphi_{a_{t+1}}=\frac{\max_{a_{t+1}} Q(S_{t+1},a_{t+1};\omega^{-})}{\sum_{a_{t+1}} Q(S_{t+1},a_{t+1};\omega^{-})}$$

$\sigma_{a_{t+1}}$ is obtained using the D3QN:

$$\sigma_{a_{t+1}}=\frac{Q(S_{t+1,}\underset{a_{t+1}}{arg max} Q(S_{t+1},a_{t+1};\omega);\omega^{-})}{\sum_{a_{t+1}} Q(S_{t+1,}\underset{a_{t+1}}{arg max} Q(S_{t+1},a_{t+1};\omega);\omega^{-})}$$

By using the adaptive dynamic weight *p*, we seek to balance the target Q-values estimated by two methods, making the approximate target Q-value closer to an unbiased estimator. The Q-value function *Q* (*S_t_*, *a_t_*) represents the expected cumulative return obtained by selecting an action *a_t_* in state *S_t_* and following a certain policy. To estimate the Q-value for the current state *S_t_*, we add the immediate reward of taking action *a_t_* to the target Q-value *Q* (*S_t+1_*, *a_t+1_*). Ultimately, we derive the Q-value function:

$$Q(S_{t},a_{t}) = r +\gamma Q(S_{t+1},a_{t+1})$$

Here,$r$ is the reward obtained after performing the action in state *S_t_*, and $\gamma$ is the discount factor. Additionally, we use clinical expertise and patient outcomes to estimate the target Q-value function and guide the agent. The Q-value function based on clinical expertise is:

$Q^{clin}(S_{t}, a_{t}^{clin}) =r +\gamma Q^{clin}(S_{t+1},a_{t+1}^{clin} ;\omega^{-})$

Therefore, if the SOFA score <*n*, we will use the Q-value function based on clinical expertise; otherwise, we will use the new Q-value function. The Q-value function for the WD3QNE algorithm is

$$Q^{WD3QNE}=\left\{ \begin{aligned} Q^{clin}(S_{t},a_{t}^{clin}) if SOFA<n \\ Q(S_{t},a_{t}) otherwise \end{aligned} \right.$$

where *n* is a hyperparameter, set to 5.

The neural network includes an input layer, a hidden layer with 256-dimensional fully connected layers, a hidden layer with 128-dimensional fully connected layers, a dueling layer (advantage layer and value layer), and an output layer. All hidden layers are activated using rectified linear units (ReLUs). The decision-making neural network parameters are updated using gradient descent. The data analysis process is illustrated in Supplementary Figure 1.


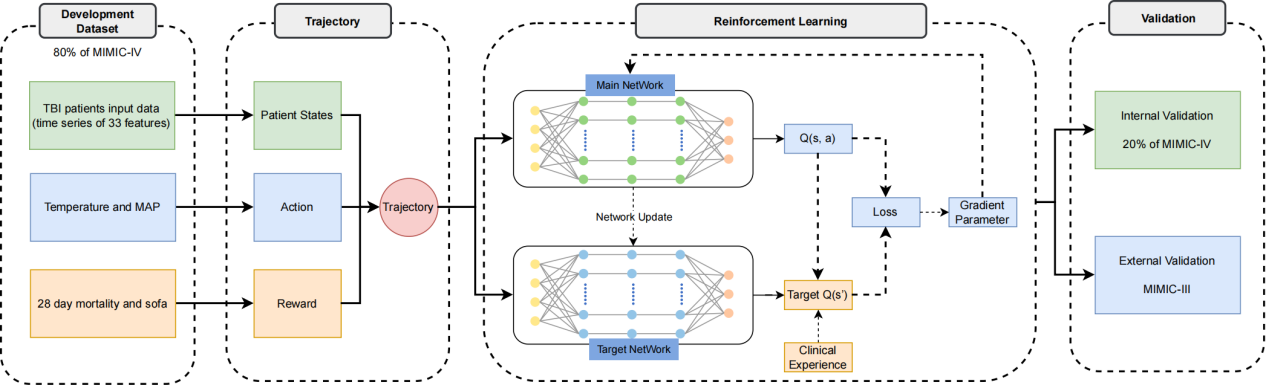


**Figure 1** flowchart of analysis

1. Watkins CJCH, Dayan P. Q-learning. Mach Learn 1992 May;8(3–4):279–292. doi: 10.1007/BF00992698

2. Annasamy RM, Sycara K. Towards Better Interpretability in Deep Q-Networks. AAAI 2019 Jul 17;33(01):4561–4569. doi: 10.1609/aaai.v33i01.33014561

3. Fu J. Deep Q-Networks for Accelerating the Training of Deep Neural Networks. arXiv; 2017. doi: 10.48550/arXiv.1606.01467

4. Mnih V, Kavukcuoglu K, Silver D, Rusu AA, Veness J, Bellemare MG, Graves A, Riedmiller M, Fidjeland AK, Ostrovski G, Petersen S, Beattie C, Sadik A, Antonoglou I, King H, Kumaran D, Wierstra D, Legg S, Hassabis D. Human-level control through deep reinforcement learning. Nature 2015 Feb 26;518(7540):529–533. doi: 10.1038/nature14236

5. Van Hasselt H, Guez A, Silver D. Deep Reinforcement Learning with Double Q-Learning. AAAI 2016 Mar 2;30(1). doi: 10.1609/aaai.v30i1.10295

6. Wang Z, Schaul T, Hessel M, van Hasselt H, Lanctot M, de Freitas N. Dueling Network Architectures for Deep Reinforcement Learning.

7. Fu Y, Shen Y, Tang L. A Dynamic Task Allocation Framework in Mobile Crowd Sensing with D3QN. Sensors 2023 Jul 1;23(13):6088. doi: 10.3390/s23136088
